# Synaptic vesicle exocytosis and increased cytosolic calcium are both necessary but not sufficient for activity‐dependent bulk endocytosis

**DOI:** 10.1111/jnc.13132

**Published:** 2015-05-14

**Authors:** Andrew Morton, Jamie R. K. Marland, Michael A. Cousin

**Affiliations:** ^1^ Centre for Integrative PhysiologyUniversity of EdinburghHugh Robson BuildingGeorge SquareEdinburghScotland

**Keywords:** calcium, dynamin, endocytosis, exocytosis, presynapse, vesicle

## Abstract

Activity‐dependent bulk endocytosis (ADBE) is the dominant synaptic vesicle (SV) endocytosis mode in central nerve terminals during intense neuronal activity. By definition this mode is triggered by neuronal activity; however, key questions regarding its mechanism of activation remain unaddressed. To determine the basic requirements for ADBE triggering in central nerve terminals, we decoupled SV fusion events from activity‐dependent calcium influx using either clostridial neurotoxins or buffering of intracellular calcium. ADBE was monitored both optically and morphologically by observing uptake of the fluid phase markers tetramethylrhodamine‐dextran and horse radish peroxidase respectively. Ablation of SV fusion with tetanus toxin resulted in the arrest of ADBE, but had no effect on other calcium‐dependent events such as activity‐dependent dynamin I dephosphorylation, indicating that SV exocytosis is necessary for triggering. Furthermore, the calcium chelator EGTA abolished ADBE while leaving SV exocytosis intact, demonstrating that ADBE is triggered by intracellular free calcium increases outside the active zone. Activity‐dependent dynamin I dephosphorylation was also arrested in EGTA‐treated neurons, consistent with its proposed role in triggering ADBE. Thus, SV fusion and increased cytoplasmic free calcium are both necessary but not sufficient individually to trigger ADBE.

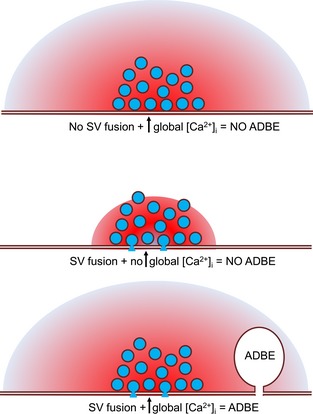

Activity‐dependent bulk endocytosis (ADBE) is the dominant synaptic vesicle (SV) endocytosis mode in central nerve terminals during intense neuronal activity. To determine the minimal requirements for ADBE triggering, we decoupled SV fusion events from activity‐dependent calcium influx using either clostridial neurotoxins or buffering of intracellular calcium. We found that SV fusion and increased cytoplasmic free calcium are both necessary but not sufficient to trigger ADBE.

**Abbreviations used**
[Ca^2+^]_i_: intracellular free calcium
ADBE: activity‐dependent bulk endocytosis
AM: acetoxymethylester
CGN: cerebellar granule neuron
CME: clathrin‐mediated endocytosis
HRP: horse radish peroxidase
SV: synaptic vesicle
sybII: synaptobrevin II
SypHy: synaptophysin‐pHlourin
TMR‐dextran: tetramethylrhodamine‐dextran
TnTx: tetanus toxin
BAPTA: 1,2‐bis(o‐aminophenoxy)ethane‐N,N,N′,N′‐tetraacetic acid

Neurotransmission depends on the efficient coupling of activity‐dependent calcium influx to the fusion of neurotransmitter‐containing synaptic vesicles (SVs). Central nerve terminals have a defined anatomy that facilitates this process. Each nerve terminal contains at least one active zone, which has a high concentration of both voltage‐sensitive calcium channels and SVs docked at the plasma membrane (Sudhof 2013). Opening of calcium channels on action potential invasion creates a localised microdomain of high intracellular free calcium ([Ca^2+^]_i_) sufficient to trigger SV exocytosis after binding to the low affinity calcium sensor synaptotagmin 1 (Sudhof 2012, 2013).

Following exocytosis, the efficient retrieval and recycling of SVs are critical for the maintenance of neurotransmission. Central nerve terminals contain a variety of endocytosis modes which facilitate SV retrieval across the whole physiological range. Single action potentials trigger an ultrafast form of SV endocytosis that occurs immediately adjacent to the active zone (Watanabe *et al*. 2013a,b), whereas trains of low frequency action potentials trigger clathrin‐mediated endocytosis (CME). CME is the dominant endocytosis mode during mild stimulation (Granseth *et al*. 2006); however, during more intense activity, it becomes saturated (Sankaranarayanan and Ryan 2000), potentially because of a finite pool of clathrin molecules (Lopez‐Murcia *et al*. 2014). Under these intense stimulation conditions, additional SV retrieval capacity is provided by activity‐dependent bulk endocytosis (ADBE), which invaginates large areas of plasma membrane to form bulk endosomes direct from the plasma membrane (Clayton and Cousin 2009; Cousin 2009). SVs are then generated from these endosomes to replenish the recycling SV pool (Clayton and Cousin 2009).

ADBE is the dominant SV endocytosis mode during intense stimulation and is triggered by a specific activity threshold (Clayton *et al*. 2008; Wenzel *et al*. 2012). This suggests that activation is because of either a critical level of SV insertion into the plasma membrane or increased [Ca^2+^]_i_. Previous studies have concentrated on the latter possibility, with a key role identified for the dephosphorylation of the large GTPase dynamin I (Clayton and Cousin 2009). In this model, intense stimulation activates the calcium‐dependent protein phosphatase calcineurin to dephosphorylate dynamin I, resulting in the recruitment of the endocytosis protein syndapin I (Clayton *et al*. 2009). Calcineurin is therefore proposed to be the calcium trigger for ADBE (Clayton *et al*. 2009; Wu *et al*. 2009).

In this study, we tackled key unaddressed questions regarding the molecular requirements for ADBE triggering in central nerve terminals. By decoupling SV fusion events from activity‐dependent calcium influx, we demonstrate that both SV exocytosis and [Ca^2+^]_i_ increases outside the active zone microdomain are necessary but not sufficient for the triggering of ADBE.

## Materials and methods

### Materials

FM1‐43, tetramethylrhodamine‐dextran (TMR‐dextran), penicillin/streptomycin, phosphate‐buffered salts, and Minimal Essential Medium were obtained from Invitrogen. Foetal calf serum was from Biosera. The dynamin I phospho‐specific Ser‐774 antibody was from AbD Serotec, the synaptophysin antibody was from Synaptic Systems and the synaptobrevin II antibody was from Nventa Biopharmaceuticals. Glutaraldehyde and osmium tetroxide were from Agar Scientific. Tetanus toxin (TnTx), BAPTA‐AM (1,2‐bis(o‐aminophenoxy)ethane‐N,N,N′,N′‐tetraacetic acid) and EGTA‐AM were from Calbiochem. Fura‐2 was from Anaspec. Synaptophysin‐pHluorin (sypHy) was a gift from Prof. Leon Lagnado (University of Sussex). All other reagents were from Sigma.

### Preparation of cerebellar granule neurons

Primary cultures of cerebellar granule neurons (CGNs) were prepared from the cerebella of 7 day old Sprague Dawley rat pups of either sex (Anggono *et al*. 2006). Pups were sourced from an in‐house breeding facility and were killed using schedule one procedures according to UK Home Office Regulations. Where indicated cultures were incubated with 2 nM TnTx for 24 h in culture medium prior to commencing experiments. For all experiments cultures were removed from culture medium into incubation medium [in mM: 170 NaCl, 3.5 KCl, 0.4 KH_2_PO_4_, 20 TES (*N*‐tris[hydroxy‐methyl]‐methyl‐2‐aminoethane‐sulphonic acid), 5 NaHCO_3_, 5 glucose, 1.2 Na_2_SO_4_, 1.2 MgCl_2_, 1.3 CaCl_2_, pH 7.4)] for 10 min before commencing experiments. In certain experiments a low calcium buffer was used which had an identical composition to incubation buffer with the exception of 10 mM MgCl_2_, 100 μM CaCl_2_ and 50 μM EGTA.

### Western blotting

Cultures were lysed with sample buffer (67 mM sodium dodecyl sulphate, 2 mM EDTA, 9.3% glycerol, 12% β‐mercaptoethanol, bromophenol blue, 67 mM Tris, pH 6.8) either at rest or immediately after a train of 800 action potentials (80 Hz). Lysate was quickly removed and boiled for subsequent analysis by sodium dodecyl sulphate polyacrylamide gel electrophoresis and western blotting. Synaptobrevin II and phospho‐dynamin antibodies were used at a dilution of 1 : 1000, whereas the synaptophysin antibody was used at 1 : 4000. The intensity of the signal from either the synaptobrevin II or phospho‐dynamin blots was normalised against the amount of synaptophysin on the same blot.

### Fluorescent calcium imaging

Cultures were loaded with 10 μM Fura‐2‐ AM in incubation buffer for 30 min at 23°C. Cells were washed three times and mounted in a Warner Instruments field stimulation imaging chamber (RC‐21BRFS) on a Nikon Diaphot‐TMD inverted microscope. Images were acquired using alternate 340 nm and 380 nm excitation with 505 nm emission using a × 20 air objective and a Hamamatsu Orca‐ER CCD camera (Japan). Regions of interest were placed over granule cell neurites and their average pixel intensity values were calculated across each image sequence using the Time Series Analyser plugin from Image J (NIH). Data acquired at both 340 and 380 nm were expressed as a ratio and calibrated using the equation provided by (Grynkiewicz *et al*. 1985), where R_min_ was obtained by addition of incubation medium supplemented with 1 mM EGTA and 10 μM ionomycin, R_max_ by subsequent addition of saturating CaCl_2_ (5 mM) with the Kd for fura‐2 assumed to be 224 nM.

### Fluorescent imaging of SV recycling using sypHy

CGNs were transfected with the genetic reporter sypHy (Granseth *et al*. 2006) using calcium phosphate precipitation between 4 and 6 days *in vitro* (Anggono *et al*. 2006). Transfected neurons were then imaged after 72 h using a × 40 oil immersion objective and 480 nm excitation and > 525 nm emission on a Zeiss Axio‐Observer A1 inverted epi‐fluorescence microscope with a AxioCam MRm Rev.3 digital camera (Zeiss Ltd, Cambridge, UK). The sypHy response was recorded before and during a challenge with a train of 800 action potentials delivered at 80 Hz (to visualise evoked SV fusion). After recording for a further 3 min, incubation medium supplemented with 50 mM NH_4_Cl (NaCl removed to maintain osmolarity) was perfused into the chamber to reveal the remaining quenched sypHy fluorescence. The fluorescence readout from defined regions of interest from transfected neurons was recorded and normalised to the peak sypHy response in the presence of NH_4_Cl.

### Fluorescent imaging of ADBE using TMR‐dextran

Uptake of TMR‐dextran (40 kDa) was monitored (Clayton *et al*. 2008). After resting in incubation medium for 10 min cultures were stimulated with a train 800 action potentials (80 Hz 10 s) in the presence of 50 μM TMR‐dextran. For EGTA‐AM and BAPTA‐AM experiments cultures were incubated with 100 μM of these chelators [or a dimethylsulfoxide (DMSO) control] for 30 min prior to loading. ADBE triggering was determined by the number of TMR‐dextran puncta in a defined field of view using a 20 ×  air objective at 550 nm excitation and > 575 nm emission on a Zeiss Axio‐observer A1 inverted epi‐fluorescence microscope with a Zeiss AxioCam MRm Rev.3 digital camera. The number of TMR‐dextran puncta per field was quantified using an automated non‐interactive analysis pipeline developed in FIJI (Schindelin *et al*. 2012). The image processing steps are listed below, with reference to the specific FIJI functions and parameters indicated in parentheses: 1. Background subtraction (Subtract background; rolling ball radius: 50 pixels) 2. Edge detection (Find edges) 3. Automated thresholding (Auto threshold; MaxEntropy method) 4. Particle counting (Analyze particles; particle size: 0.2–1 μm; include holes). The resulting particle count data from each image were collated in Microsoft Excel to generate the mean number of puncta per field. All data are presented as the difference between evoked and resting TMR‐dextran uptake.

### Fluorescent imaging of SV recycling using FM1‐43

SV turnover was monitored using an S2/S1 protocol (Clayton *et al*. 2010). Briefly, after a 10 min rest in incubation buffer cultures were mounted in a Warner imaging chamber on a Zeiss Axio‐Observer A1 inverted epi‐fluorescence microscope. FM1‐43 (10 μM) was loaded using a train of 800 action potentials at 80 Hz with immediate dye washout following stimulation. Cultures were continuously perfused with incubation buffer for 10 min before FM1‐43 unloading was evoked with a train of 800 action potentials (80 Hz). This provides an estimate of the total number of SVs turned over during stimulation (ΔS1). After a 10 min rest period the S1 protocol was repeated (S2 loading and unloading). Thus, for any selected nerve terminal, the ΔS2 response has a matched individual internal control (ΔS1). EGTA‐AM, BAPTA‐AM (both 100 μM) or an equal volume of vehicle (DMSO) was incubated with cultures after the S2 load for 30 min before the S2 unloading step. Results are represented as the average ΔS2/ΔS1. Dye unloading was visualised using a 20 ×  air objective at 480 nm excitation and > 510 nm emission with a Zeiss AxioCam MRm Rev.3 digital camera.

### Morphological analysis of SV endocytosis modes

Cultures were fixed and processed for electron microscopy (Clayton *et al*. 2008). Cultures were either incubated with DMSO (vehicle), BAPTA‐AM or EGTA‐AM (both 100 μM) in incubation medium for 30 min before stimulation with a train of 800 action potentials in the presence of 10 mg/mL horse radish peroxidase (HRP). TnTx‐treated cultures were subjected to the same stimulus and loading, but without the 30 min incubation period. Cultures were then immediately fixed in 2% glutaraldehyde in phosphate‐buffered saline. After washing with 100 mM Tris (pH 7.4), cultures were exposed to 0.1% diaminobenzidine and 0.2% H_2_O_2_ in 100 mM Tris until colour developed. Cultures were then washed with 100 mM Tris, stained with 1% osmium tetroxide for 30 min and then dehydrated and embedded in TAAB LEMIX resin (TAAB Laboratories Equipment, Aldermaston, UK). Ultrathin sections were cut and mounted on grids, stained with uranyl acetate and lead citrate and viewed using a Philips BioTWIN CM120 transmission electron microscope Intracellular structures that were < 100 nm in diameter were arbitrarily designated to be SVs, whereas larger structures were considered endosomes.

### Statistical analysis

In experiments using fluorescence imaging the experimental n is the number of coverslips examined. For fura‐2 and FM1‐43 experiments at least 90 regions of interest were sampled per coverslip. For sypHy experiments at least 30 regions of interest were sampled per coverslip. For TMR‐dextran experiments at least five fields of view were sampled per coverslip. A students *t* test was used for comparisons between two groups, whereas a one‐way anova was used for comparison between multiple groups as indicated in the figure legends.

## Results

### Extracellular calcium influx is essential for activation of ADBE

**Figure 1:**
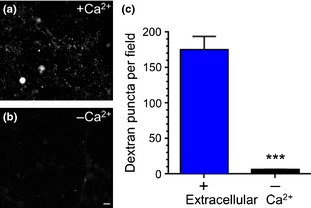
Calcium influx is essential for activity‐dependent bulk endocytosis (ADBE). Cerebellar granule neurons were incubated with tetramethylrhodamine‐dextran (TMR‐dextran) (50 μM) during a train of 800 action potentials (80 Hz) in the presence or absence of extracellular calcium. (a and b) Representative images of TMR‐dextran loading in either plus calcium (a) or low calcium (b) buffer. Scale bar represents 1 μm. (c) Quantification of the number of TMR‐dextran puncta per field of view in either the presence (blue bars) or absence (black bars) of extracellular calcium ± SEM. ***p = 0.0008, Student's *t*‐test, *n* = 3 coverslips per condition.

Intense neuronal activity triggers ADBE; however, basic questions relating to which initial events are either necessary or sufficient to activate this endocytosis mode have still to be addressed. The most basic question is whether calcium influx is required for triggering of ADBE during high frequency stimulation. To address this we visualised ADBE by monitoring the uptake of a large molecular weight fluid phase marker conjugated to a fluorescent molecule (40 kDa TMR‐dextran) into primary cultures of CGNs. TMR‐dextran uptake was monitored in either the presence or absence of extracellular calcium during a train of 800 action potentials delivered at 80 Hz. A robust uptake of TMR‐dextran was observed in the presence of extracellular calcium indicating triggering of ADBE (Fig. 1a). In contrast negligible uptake was observed in the absence of calcium (Fig. 1b and c). Thus, the presence of extracellular calcium during intense neuronal activity is essential for the triggering of ADBE.

### SV fusion is necessary for ADBE triggering

**Figure 2:**
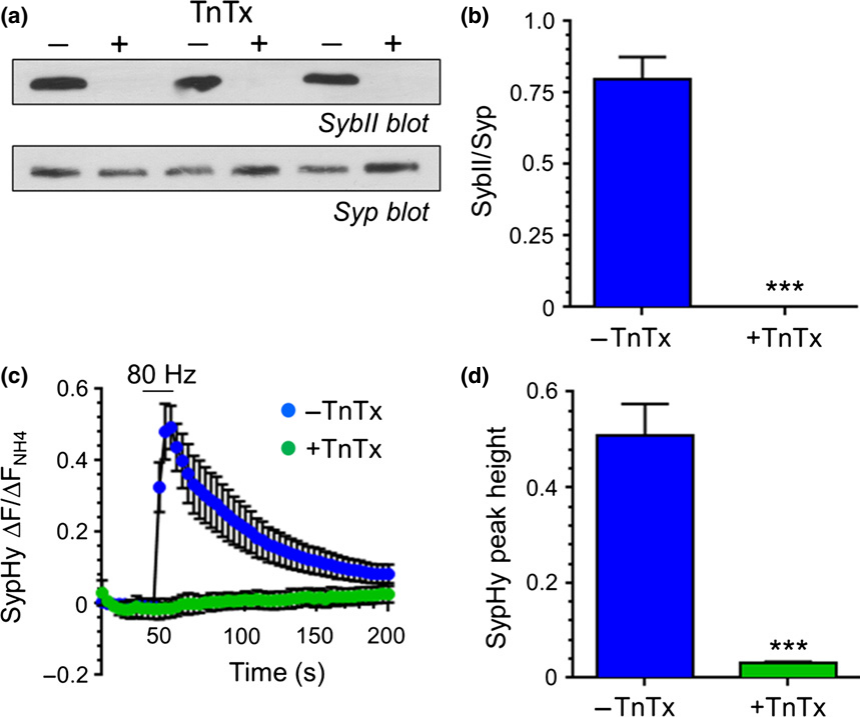
Tetanus toxin (TnTx) ablates activity‐dependent SV exocytosis. Cultures were preincubated either with or without 2 nM TnTx for 24 h. (a) Representative immunoblots of cerebellar granule neuron lysates probed with synaptobrevin II (SybII) antibodies. The same blot was reprobed with synaptophysin (Syp) antibodies. (b) Quantification of sybII levels after normalisation against Syp ± SEM. SybII levels were zero in each experiment. ***p = 0.0005, Student's *t*‐test, *n* = 3 independent experiments per condition. (c and d) Cultures were transfected with the genetic reporter synaptophysin‐pHluourin (sypHy) and stimulated with a train of 800 action potentials (80 Hz). They were subsequently exposed to alkaline buffer to reveal the total sypHy fluorescence. (c) Graph displays the mean ΔF/F_0_ time course for sypHy ± SEM in either untreated (blue circles) or treated (green circles) neurons normalised to the total sypHy response in alkaline buffer. Bar indicates period of stimulation. (d) Bar graph displays the maximal evoked ∆F of sypHy for untreated (blue bars) or treated (green bars) neurons normalized to the total SV pool (revealed by the alkaline buffer) ± SEM. ***p = 0.0006, Student's *t*‐test, *n* = 3 independent experiments per condition.

Extracellular calcium influx initiates a cascade of intracellular events in central nerve terminals, including SV exocytosis. We next determined whether the fusion of SVs with the plasma membrane was necessary for ADBE triggering. This was achieved by incubating cultures for 24 h with the clostridial neurotoxin TnTx (2 nM) which digests the v‐SNARE synaptobrevin II (sybII) (Schiavo *et al*. 1992). After 24 h the digestion of sybII was complete, as confirmed by quantitative western blotting of culture lysates (Fig. 2a and b). To confirm that TnTx treatment eliminated SV exocytosis, we employed the fluorescent genetic reporter sypHy (Granseth *et al*. 2006). SypHy has a pH‐sensitive green fluorescent protein moiety inserted into an intraluminal loop of the integral SV protein synaptophysin. It is highly fluorescent at neutral pH, but its fluorescence is quenched by acidic environments such as the SV interior. Thus, the evoked fusion of SVs can be visualised as an increase in fluorescence on action potential stimulation, whereas endocytosis can be viewed as a fluorescence decrease [as SV endocytosis is rate limiting rather than SV acidification (Sankaranarayanan and Ryan 2001)]. When cultures were stimulated with a train of 800 action potentials a characteristic sypHy response was observed, with a rapid increase during stimulation (reflecting SV exocytosis) followed by a slower decrease (reflecting SV endocytosis, Fig. 2c). In contrast, no sypHy response was elicited by the same stimulation protocol in TnTx‐treated cultures (Fig. 2c and d). Thus, TnTx treatment abolishes evoked SV fusion in our culture system.

**Figure 3:**
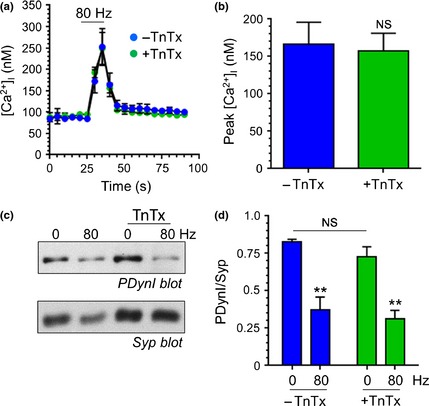
Tetanus toxin (TnTx) does not affect calcium influx or dynamin I dephosphorylation. Cultures were preincubated either with or without 2 nM TnTx for 24 h. (a and b) Cultures were loaded with fura‐2‐AM (10 μM) and then stimulated with a train of 800 action potentials (80 Hz). (a) Representative graphs display the fura‐2 response from TnTx‐treated (blue circles) and ‐untreated (green circles) cultures displayed as evoked increase in [Ca^2+^]_i_ ± SEM. Bar indicates period of stimulation. (b) Quantification of the peak evoked [Ca^2+^]_i_ response ± SEM for untreated (blue) or treated (green) cultures. Student's *t*‐test, *p* = 0.82, *n* = 3 independent experiments per condition. (c) Cultures treated with or without TnTx were either stimulated with a train of 800 action potentials (80 Hz) or left to rest for 10 s. Cultures were then immediately lysed in SDS sample buffer. Representative immunoblots of cerebellar granule neuron lysates probed with dynamin I phospho‐Ser774 antibodies (PDynI). The same blot was then reprobed with synaptophysin (Syp) antibodies. (d) Quantification of PDynI levels after normalisation against Syp (PDynI/Syp) ± SEM in untreated (blue) or untreated (green) cultures. **p <* *0.01 for the evoked dephosphorylation in either TnTx‐treated or non‐treated lysates, one‐way AVOVA,* n *=* *3 independent experiments per condition.

We next confirmed that activity‐dependent calcium influx was unaffected by TnTx treatment by monitoring intracellular free calcium ([Ca^2+^]_i_) using the fluorescent probe fura‐2‐AM (Grynkiewicz *et al*. 1985; Cousin *et al*. 1995). TnTx had no effect on either baseline [Ca^2+^]_i_ levels or the evoked [Ca^2+^]_i_ response (Fig. 3a and b). We next examined whether activity‐dependent dynamin I dephosphorylation was affected by TnTx, as ADBE is proposed to be triggered by this event (Clayton *et al*. 2009). This was achieved by using phospho‐specific antibodies to the key residue Ser‐774 (Tan *et al*. 2003). Both TnTx‐treated and ‐untreated cultures displayed a characteristic activity‐dependent dephosphorylation on stimulation with a train of 800 action potentials (Fig. 3c). Quantitative analysis confirmed the lack of effect of TnTx on activity‐dependent dynamin I dephosphorylation (Fig. 3d). Thus, TnTx treatment decouples SV fusion from both activity‐dependent calcium influx and dynamin I dephosphorylation.

**Figure 4:**
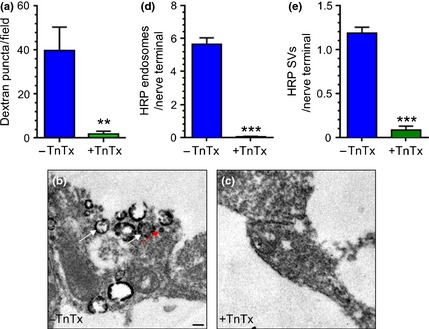
Tetanus toxin (TnTx) abolishes activity‐dependent bulk endocytosis (ADBE). Cultures were preincubated either with or without 2 nM TnTx for 24 h. (a) Cultures were loaded with tetramethylrhodamine‐dextran (TMR‐dextran) (50 μM) during stimulation with a train of 800 action potentials (80 Hz). Quantification of the number of evoked TMR‐dextran puncta per field of view in cultures treated with (green bars) or without (blue bars) TnTx ± SEM. **p = 0.008, Student's *t*‐test, *n* = 5 coverslips per condition. (b–e) Cultures were loaded with 10 mM horse radish peroxidase (HRP) during stimulation with a train of 800 action potentials (80 Hz). (b and c) Representative images of nerve terminals after HRP loading in cultures either treated with (+) or without (−) TnTx. Scale bar indicates 200 nm. White arrow indicates HRP‐endosomes, red arrow indicates HRP‐SVs. (c and d) Quantification of the number of HRP‐labelled endosomes (d) or HRP‐labelled SVs (e) ± SEM per nerve terminal in untreated (blue bars) or treated (green bars) cultures. Student's *t*‐test, ***p < 0.0001, *n* = 3 independent experiments per condition.

We next determined whether SV fusion was necessary for ADBE by monitoring TMR‐dextran uptake in TnTx‐treated cultures. TnTx treatment abolished TMR‐dextran uptake evoked by 800 action potentials delivered at 80 Hz (Fig. 4a). In contrast untreated cultures displayed a robust uptake (Fig. 4a). To monitor ADBE via a different approach, uptake of the fluid phase marker HRP was assessed. ADBE and CME can be monitored in parallel using this technique with activation of ADBE reported as an increase in HRP‐labelled endosomes and CME reported as an increase in HRP‐labelled SVs (Clayton *et al*. 2008). Cultures that were stimulated with a train of 800 action potentials (80 Hz) displayed nerve terminals that contained both HRP‐labelled endosomes and SVs, indicating triggering of both ADBE and CME (Fig. 4b). However, when TnTx‐treated cultures were examined there was a complete absence of HRP‐labelled endosomes and a large reduction in the number of HRP‐labelled SVs, indicating a lack of activity‐dependent endocytosis in these cultures (Fig. 4c–e). Thus, SV fusion is necessary for the triggering of ADBE.

### ADBE is triggered by increased cytoplasmic calcium outside the active zone

SV exocytosis is triggered by highly localised calcium influx generated via clustering of voltage‐sensitive calcium channels at the active zone (Sudhof 2013). This clustering creates a microdomain of highly concentrated calcium required for the triggering of SV fusion by the low affinity calcium sensor synaptotagmin I (Sudhof 2012). We next determined whether this localised calcium influx or a summation of cytoplasmic calcium outside the active zone was required to trigger ADBE. To achieve this, we utilised two high affinity intracellular calcium chelators that have different rates of calcium binding, BAPTA and EGTA (Adler *et al*. 1991). BAPTA binds calcium with an approximately 100 times faster on‐rate than EGTA and thus is able to prevent the formation of microdomain calcium at the active zone, whereas EGTA cannot (Adler *et al*. 1991; Schneggenburger and Neher 2005).

**Figure 5:**
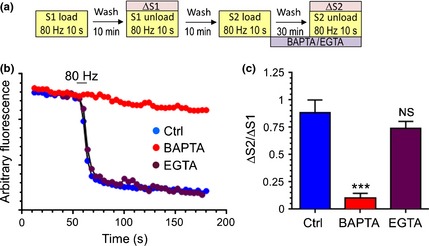
SV exocytosis is arrested by BAPTA‐AM but not by EGTA‐AM. (a) Schematic of the experimental design. Cultures were loaded with 10 μM FM1‐43 during a train of 800 action potentials (80 Hz). After a 10 min rest period FM1‐43 was unloaded with a second 800 action potential stimulus (S1). After a 10 min rest period, the same loading/unloading protocol was performed again, with the exception being that cultures were incubated with either BAPTA‐AM, EGTA‐AM (both 100 μM) or dimethylsulfoxide (DMSO) (Ctrl) for 30 min directly after S2 loading. (b) Representative traces showing the S2 unloading profiles of Ctrl (blue circles), BAPTA‐AM (red circles) and EGTA‐AM (purple circles) ‐treated cultures. (c) Quantification of the extent of FM1‐43 unloading at both S1 (ΔS1) and S2 (ΔS2) presented as the ratio ΔS2/ΔS1 ±  SEM. ***p <0.001, One‐way‐anova,* n *=* *3 Ctrl, *n *=* *4 EGTA‐AM,* n *=* *5 BAPTA‐AM.

We first confirmed these chelators were acting as predicted in our system. To achieve this we incubated cultures with acetoxymethylester (AM) versions of either BAPTA or EGTA and determined their effect on SV exocytosis using an S2/S1 assay with the fluorescent dye FM1‐43 (Clayton *et al*. 2010). In this protocol SV turnover was evoked (800 action potentials, 80 Hz) to load FM1‐43 into retrieving SVs. After a 10 min rest period SV turnover was stimulated by an identical stimulus and SV exocytosis was visualised as an activity‐dependent loss of FM1‐43 fluorescence (ΔS1). This protocol was then repeated in the same nerve terminals with the only change being incubation with either BAPTA‐AM or EGTA‐AM (both 100 μM) for 30 min after loading of FM1‐43 (Fig. 5a). This allows a determination of the effect of these chelators on SV exocytosis after normalising to an internal control (ΔS2/ΔS1). Vehicle‐treated cultures displayed a ΔS2/ΔS1 ratio of approximately 1, indicating no defect on SV exocytosis at S2 (Fig. 5b). BAPTA‐AM‐treated cultures displayed an almost complete ablation of SV exocytosis at S2, whereas SV exocytosis in EGTA‐AM‐treated cultures was not significantly different to control (Fig. 5b and c). Thus, BAPTA‐AM abolishes SV exocytosis by blocking formation of the calcium microdomain, whereas the slower binding EGTA‐AM cannot.

**Figure 6:**
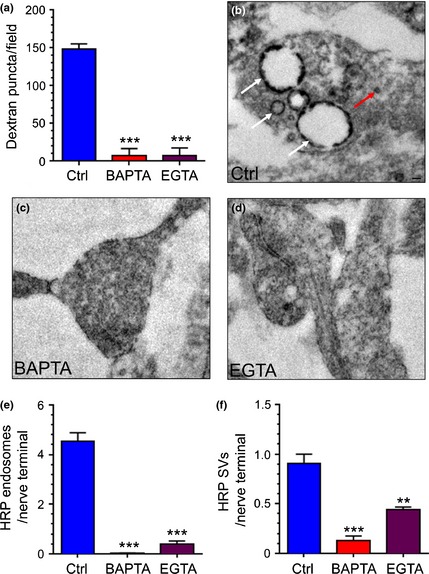
Activity‐dependent bulk endocytosis (ADBE) is abolished by both BAPTA‐AM and EGTA‐AM. Cultures were preincubated with BAPTA‐AM, EGTA‐AM (both 100 μM) or DMSO (vehicle control) for 30 min before loading with tetramethylrhodamine‐dextran (TMR)‐dextran (50 μM) during stimulation with a train of 800 action potentials (80 Hz). (a) Quantification of the number of evoked TMR‐dextran puncta per field of view in cultures treated with either DMSO (Ctrl, blue bars) BAPTA‐AM (red bars) or EGTA‐AM (purple bars) ± SEM. ***p < 0.001, one‐way anova,* n *=* *3 coverslips per condition. (b–f) DMSO, BAPTA‐AM or EGTA‐AM‐treated cultures were loaded with horse radish peroxidase (HRP) during stimulation with a train of 800 action potentials (80 Hz). (b) Representative images of nerve terminals after HRP loading in DMSO (Ctrl, B), BAPTA‐AM (c) or EGTA‐AM (d) ‐treated cultures. Scale bar indicates 100 nm. White arrow indicates HRP‐endosomes. Red arrow indicates HRP SVs. (e and f) Quantification of the number of HRP‐labelled endosomes (e) or HRP‐labelled SVs (f) ± SEM per nerve terminal in either Ctrl (blue bars), BAPTA‐AM (red bars) or EGTA‐AM (purple bars) –treated cultures. One‐way anova, ***p <* *0.001, **p <* *0.01, *n *=* *3 independent experiments per condition.

We next determined the effect of both BAPTA‐AM and EGTA‐AM on ADBE by monitoring TMR‐dextran uptake in response to a train of 800 action potentials (80 Hz). Vehicle‐treated cultures displayed a robust uptake of TMR‐dextran, indicative of triggering of ADBE (Fig. 6a). BAPTA‐AM treatment ablated TMR‐dextran uptake, confirming the essential requirement for SV fusion (Fig. 6a). Interestingly, TMR‐dextran uptake was also eliminated in cultures treated with EGTA‐AM (Fig. 6a), suggesting that [Ca^2+^]_i_ increases outside the active zone are required to trigger ADBE. To confirm this requirement we also examined the effect of EGTA‐AM on HRP uptake into nerve terminals. Vehicle‐treated cultures displayed a large increase in both HRP‐labelled endosomes and HRP‐labelled SVs on stimulation with 800 action potentials (80 Hz, Fig. 6b). BAPTA‐AM treatment abolished the appearance of both HRP‐labelled endosomes and SVs, indicating arrest of both endocytosis modes (Fig. 6c and e). Importantly the formation of HRP‐labelled endosomes in EGTA‐AM treated cultures was abolished, confirming arrest of ADBE with this treatment (Fig. 6d and e). This inhibition was not as pronounced for HRP‐labelled SVs, suggesting CME is not as tightly coupled to [Ca^2+^]_i_ as ADBE (Fig. 6f). Thus, activity‐dependent [Ca^2+^]_i_ increases outside the active zone are necessary to trigger ADBE.

**Figure 7:**
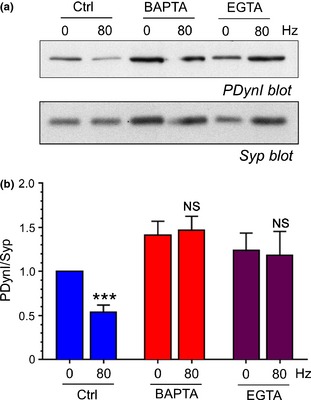
Dynamin I dephosphorylation is arrested by BAPTA‐AM and EGTA‐AM. Cultures were preincubated with BAPTA‐AM, EGTA‐AM (both 100 μM) or DMSO (vehicle control) for 30 min. They were then either stimulated with a train of 800 action potentials (80 Hz) or left to rest for 10 s. Cultures were then immediately lysed in SDS sample buffer. (a) Representative immunoblots of cerebellar granule neuron lysates probed with either dynamin I phospho‐Ser774 (PDynI) or synaptophysin (Syp) antibodies. (b) Quantification of PDynI levels after normalisation against Syp (PDynI/Syp) ± SEM. Blue bars represent Ctrl, red bars BAPTA‐AM and purple bars EGTA‐AM, ***p < 0.001, Student's *t*‐test, *n* = 9 independent experiments per condition.

The activity‐dependent dephosphorylation of dynamin I by calcineurin correlates with the triggering of ADBE (Clayton *et al*. 2009). If dynamin I dephosphorylation was the trigger for ADBE we would predict that its dephosphorylation would also be arrested by EGTA‐AM treatment. To test this, we monitored dynamin I phosphorylation using phospho‐specific Ser‐774 antibodies. In vehicle‐treated cultures dynamin I is dephosphorylated on stimulation with a train of 800 action potentials (80 Hz, Fig. 7). This dephosphorylation is arrested in cultures treated with either BAPTA‐AM or EGTA‐AM (Fig. 7). The inhibition of dynamin I dephosphorylation in the presence of EGTA‐AM is consistent with this event being central in the triggering of this endocytosis mode.

## Discussion

ADBE is strictly coupled to neuronal activity; however fundamental questions relating to the basic mechanisms of its activation have largely remained unaddressed. In this study, we sought to decouple calcium influx from SV fusion to determine whether both or either of these key molecular events were either necessary or sufficient for ADBE to proceed. We showed that both SV fusion and [Ca^2+^]_i_ increases outside the active zone are necessary to trigger this endocytosis mode.

Digestion of sybII by TnTx fully decoupled SV fusion from calcium influx, as evidenced by the lack of effect of the toxin on both activity‐dependent [Ca^2+^]_i_ increases and dynamin I dephosphorylation. TnTx treatment abolished ADBE, indicating an essential requirement for prior SV fusion for this SV retrieval mode. Interestingly, some HRP‐labelled SVs were present in TnTx‐treated cultures, indicating that SV retrieval still occurred albeit at a much reduced level. A similar phenotype was also observed in neuronal cultures derived from sybII knockout mice (Deak *et al*. 2004). The nature of these recycling SVs is still unclear; however, they may reflect spontaneous SV fusion and retrieval. In support, recent studies have proposed that spontaneous neurotransmission is mediated by a discrete pool of SVs (Sara *et al*. 2005; Fredj and Burrone 2009) and that this pool is defined by the presence of the TnTx‐insensitive syb isoform, sybVII (Hua *et al*. 2011; Bal *et al*. 2013).

Ideally, we also wanted to directly decouple calcium influx from SV fusion, by evoking SV exocytosis in the absence of calcium influx. We attempted this using the spider toxin α‐latrotoxin (Silva *et al*. 2009), although, using this approach, we were unable to evoke FM1‐43 unloading in the absence of extracellular calcium (data not shown). It is noted, however, that black widow spider venom (from which α‐latrotoxin is purified) evoked massive SV exocytosis and no FM1‐43 uptake at motor nerve terminals, suggesting that calcium influx is equally essential for ADBE (Henkel and Betz 1995). We also considered using hypertonic sucrose stimulation in the absence of calcium. However, this approach only mobilises the readily releasable pool (Rosenmund and Stevens 1996) and is therefore not sufficient to trigger ADBE even in the presence of extracellular calcium (Cheung *et al*. 2010).

As it was not possible to evoke SV fusion in the absence of calcium, we took a different strategy to examine the role of calcium influx in ADBE, by interfering with calcium handling in nerve terminals. We found that the slow binding calcium chelator EGTA‐AM ablated ADBE, while having no effect on SV exocytosis. Thus, both calcium influx and SV fusion are necessary but not sufficient individually to trigger ADBE. A number of studies in large atypical central nerve terminals have examined the role of localised and delocalised [Ca^2+^]_i_ increases on ‘fast’ endocytosis using capacitance analysis. This fast form may reflect ADBE, as it is calcium‐dependent (Beutner *et al*. 2001; Neves *et al*. 2001; Wu *et al*. 2005, 2009; Yamashita *et al*. 2010; Wu and Wu 2014) and clathrin‐independent (Jockusch *et al*. 2005). In support of our work, EGTA ablated this form of endocytosis (Neves *et al*. 2001; Wu *et al*. 2005). However, it is difficult to extrapolate these findings to typical small central nerve terminals because of the different geometry and the number of active zones in these nerve terminals, resulting in a different sensitively to EGTA buffering (Schneggenburger and Neher 2005). The control of SV endocytosis by [Ca^2+^]_i_ increases has also been debated in typical small central nerve terminals, with both positive and negative roles proposed (Cousin and Robinson 2000; Sankaranarayanan and Ryan 2001; Balaji *et al*. 2008; Sun *et al*. 2010; Yao *et al*. 2012). More recent studies using low frequency stimulation showed that calcium influx may control SV endocytosis in two phases, an initial acceleration followed by a slowing of endocytosis (Armbruster *et al*. 2013). Interestingly, this calcium‐dependent slowing was reversed by EGTA‐AM (Leitz and Kavalali 2011; Armbruster *et al*. 2013). Thus, [Ca^2+^]_i_ increases outside the active zone may retard CME while triggering ADBE. Our data suggest that the opposite may be true for CME, as EGTA limits the generation of HRP‐labelled SVs during high frequency stimulation.

Activity‐dependent dephosphorylation of dynamin I correlates with the triggering of ADBE (Clayton *et al*. 2009). Furthermore, over‐expression of dominant negative dynamin I phospho‐mutants, delivery of competitive peptides that block the phospho‐dependent dynamin – syndapin interaction and inhibition of the protein kinases that rephosphorylate dynamin I all arrest ADBE (Clayton *et al*. 2009, 2010). Thus, multiple threads of evidence suggest that dynamin I dephosphorylation is the activity‐dependent calcium sensor for ADBE. This agrees with our finding that EGTA‐AM arrests both ADBE and dynamin I dephosphorylation. However, recent studies have cast doubt on this model. When dynamin I expression was ablated by genomic knockout, a form of bulk fluid phase retrieval very similar to ADBE occurred at mammalian central nerve terminals (Hayashi *et al*. 2008; Wu *et al*. 2014). In contrast, siRNA‐mediated knockdown of dynamin I and III only perturbed endocytosis during high frequency stimulation, suggesting a key role in ADBE (Kononenko *et al*. 2014). A role for dynamin in endosome fission was also proposed using acute photoinactivation at *Drosophila* neuromuscular junctions (Kasprowicz *et al*. 2014). In this work, endosomes were formed but could not detach from the plasma membrane. Thus, while the essential role for dynamin I dephosphorylation in ADBE triggering is currently debated, our results are consistent with this hypothesis.

We have shown that both SV fusion and [Ca^2+^]_i_ increases outside the active zone are necessary but not individually sufficient to trigger ADBE. These essential requirements are consistent with the triggering of ADBE by high intensity stimulation, as [Ca^2+^]_i_ will only increase outside the active zone after an action potential train. This also suggests that the calcium trigger for ADBE is not located at the active zone, with calcineurin being an obvious candidate. Interestingly, a number of calcineurin substrates are localised to the reserve SV pool in addition to other endocytosis molecules (Evergren *et al*. 2004, 2007; Koh *et al*. 2007; Sundborger *et al*. 2011). Thus, ADBE may be triggered by the calcium‐dependent dephosphorylation and mobilisation of endocytic molecules that are anchored at the reserve SV pool (Shupliakov 2009; Shupliakov *et al*. 2011). A number of lines of evidence support this. First, the reserve SV pool is mobilised by the same stimuli that trigger ADBE (Cheung *et al*. 2010), indicating that it is in the correct location to sense [Ca^2+^]_i_ increases that evoke ADBE. Second, modulation of [Ca^2+^]_i_ causes the release of endocytosis molecules from SV clusters even in the absence of activity (Denker *et al*. 2011). Finally, ADBE‐derived SVs selectively replenish the reserve SV pool (Cheung *et al*. 2010), suggesting that a key role of ADBE is to restore this specific pool after its activity‐dependent mobilisation.

In summary, we have determined the basic requirements for the triggering of ADBE in central nerve terminals, with both SV fusion and calcium influx being necessary but not sufficient for the process.
